# Opinion of health professionals and drug users before the forthcoming opening of the first drug consumption room in Paris: a quantitative cross-sectional study

**DOI:** 10.1186/s12954-018-0260-8

**Published:** 2018-10-25

**Authors:** Grégoire Cleirec, Maeva Fortias, Vanessa Bloch, Virgile Clergue-Duval, Frank Bellivier, Thomas Dusouchet, Céline Debaulieu, Florence Vorspan

**Affiliations:** 1 Service de médecine addictologique, Hôpital René Muret, APHP, Sevran, France; 2 Département de Psychiatrie et de Médecine Addictologique, Hôpital Fernand Widal, APHP, Paris, France; 3 Faculté de Médecine, Université Paris Diderot, Paris, France; 4 Inserm Umrs1144 Variabilité de Réponse aux Psychotropes, Paris, France; 5 Association Gaïa, Paris, France

**Keywords:** Drug consumption room, Supervised injection facility, Intravenous drug users, Opiates, Crack/cocaine, General practitioner, Pharmacists, Emergency doctors, France

## Abstract

**Background:**

On the brink of the opening of the first French drug consumption room in Paris, the general opinion of the local involved health care professionals and drug users was not known. The objective of this study was to determine their expectations and to search for influencing factors.

**Method:**

We carried out a quantitative cross-sectional study. A multiple choice questionnaire was proposed to the surrounding willing general practitioners (GPs) and pharmacists, to the emergency doctors of Lariboisière hospital, and to the professionals of the harm reduction facilities and their drug users (PWUD). For each question, there was a choice between seven answers, from “− 3” (very negative impact) to “+ 3” (very positive impact). The influence of the characteristics of each group on its mean answers was explored by Mann-Whitney, Kruskal-Wallis, and Spearman’s tests.

**Results:**

The median expectations among the groups of responding GPs (*N* = 62), other health care professionals (*N* = 82), and PWUD (*N* = 57) were mainly positive. They thought that the drug consumption room (DCR) would improve the health of PWUD, reduce their at-risk behaviors, would not increase drug use or drug dealing in the neighborhood, and would reduce nuisance in the public space. Only the group of GPs expressed that the DCR could decrease the quietness of the neighborhood, and only the group of PWUD had higher expectations that the DCR would decrease the number of arrests and the number of violent behavior. GPs’ expectations were significantly better in terms of health improvement of PWUD and reducing their precariousness if they had a previous experience in addiction medicine (Mann-Whitney, *p* = 0.004 and *p* = 0.019), with a longer practice (Spearman’s rho, *p* = 0.021 and *p* = 0.009), and if they were currently prescribing opioid substitution treatments (Mann-Whitney, *p* = 0.030 and *p* = 0.002).

Among non-GPs, those who were working in addiction medicine centers had significantly better expectations than pharmacists, and the professionals of the local emergency department had intermediate expectations.

**Conclusions:**

Health care professionals and drug users had a positive opinion of the to-be-created Parisian drug consumption room. Experience in addiction medicine influenced positively health professionals’ expectations.

## Background

Drug consumption rooms (DCRs) are facilities operated by social workers, nurses, and medical doctors intended to provide a safe, hygienic, and therapeutic environment for the consumption of pre-obtained drugs, under professional supervision and in a non-judgmental environment [1–3]. Their primary goal is the outreach and health improvement of marginalized people who use drugs (PWUD). Their secondary goals are the reduction of drug use-related health risks (such as HIV or HCV infection, overdoses, skin and soft tissues infection or lesions secondary to drug injection), the improvement of health care of PWUD, and providing them with the opportunity to meet social workers. DCR can indirectly decrease drug-related public disorders (such as public drug use, smoking and injection devices abandoned in public space, drug-related violence, drug trafficking) [1–3]. They are also careful to coordinate their practice in the local security policy (working with mayors and law enforcement forces) in order not to promote drug use or increase drug-related crime [1, 2, 4].

The first DCR opened in Bern, Switzerland, in 1986 [1]. It was mainly intended to reduce the economic and health consequences of the HIV epidemic among PWUD and the public nuisance linked to the important local open drug scene [5]. Other facilities then opened in Germany and the Netherlands in the 1990s, and today, more than 90 DCRs have been established in nine countries [5]. Originally designed for the injection of drugs, now DCRs often also allow the inhalation of substances [1, 6].

The efficiency of DCRs regarding their different objectives has been demonstrated, although it has been argued that most of the studies originated from only two cities (Sydney and Vancouver) and that more European studies aiming to evaluate DCRs would be warranted [7]. More specifically, it has been shown that (i) DCRs reduce overdose-induced mortality [8] and increase the access to addiction treatment programs [9, 10]; (ii) DCRs reduce syringe sharing and syringe reuse and provide education on safer injection practices [11, 12], with different estimations on the extent to which they participate in reducing viral transmissions of HIV [13, 14]; (iii) DCRs do not have the adverse effect of inciting drug use, since they are not related to any increase of the number of PWUD or of the amount of drug consumed by PWUD [15]; (iv) DCRs reduce injections and injection-related waste in the public space [16] and do not increase drug-related crime, violence, and trafficking [1, 2, 4, 17, 18].

In France, the debate regarding the opening of DCRs has been going on since the 1990s [19]. Before a law passed in 2016, harm reduction programs could provide injection material (such as needles, syringes) but were not allowed to have PWUD injecting in their facilities. Furthermore, only methadone and buprenorphine oral maintenance treatment are approved in France and there is no care facility allowed to provide prescribed injectable heroin or morphine as a maintenance treatment. In 2010, the report commissioned by the Ministry of Health to the research institute INSERM on harm reduction was in favor of the opening of DCRs in France [20], but a controversy persisted [21]. The experimentation of DCR has been finally voted in January 2016, and the first DCR opened in Paris on the 10 October 2016 in the 10th district of Paris, next to North end station and adjoining Lariboisière general university hospital. This district had an open drug scene, especially with users dealing and using intravenously morphine sulphates [22].

Some studies emphasized that the acceptability of DCR depends in part of the previous assessment of the opinion of the involved people [23]. On the brink of the opening of the first French DCR, the opinion of the French drug users regarding such a facility had not been investigated. The opinion the surrounding general practitioners (GPs) had been evaluated by one study conducted in 2015, in which 61.5% of the GPs working in the neighborhood of the future DCR declared themselves in favor of its opening [24]. The opinion of others health professionals involved with PWUD, such as pharmacists, emergency room doctors, and addiction care/harm reduction professionals, was not known.

In the international literature, the opinion of PWUD regarding DCR has been extensively investigated in several countries: surveys and qualitative studies have shown that they were predominantly in favor of DCRs in Canada, Australia, Denmark, UK, and in the USA [25–30].

The opinion of the emergency doctors in Canada is also known: 74.5% of the respondents were in favor in the implementation of DCRs, and 84.6% of them would refer patients to DCR if they did exist [31].

To our knowledge, no other international study has researched the opinion of the other health professionals directly involved with PWUD regarding DCRs.

This is why, shortly before the opening of the first French DCR in Paris, we conducted this study to determine the opinion of the nearby, GPs, pharmacists, emergency doctors, harm reduction professionals, and PWUD regarding the consequences of this opening. The secondary objective was to look for factors influencing this opinion.

## Methods

We carried out a quantitative cross-sectional study.

The inclusion criterion for the GPs and the pharmacists was to work in the 9th, 10th, and 18th district of Paris (districts that are in the surroundings of the site of the DCR, and are known to hold a population of socially disadvantaged drug users). The inclusion criterion for the emergency doctors was to work in the emergency room of Lariboisière hospital (adjoining the location of the DCR). The inclusion criteria for the harm reduction professionals was to work in an addiction care or harm reduction facility of the 9th, 10th, 18th, and 19th districts. The inclusion criteria for the PWUD were to be over 18 years old (the French age of majority), to use or to have used an illegal drug and to visit a care or harm reduction facility of the area, where they were recruited.

We created a multiple choice questionnaire (MCQ) based on the positive and negative goals of the DCRs, reformulated as 14 items (see Table 1). The MCQ had 14 questions about the impact of the opening of the DCR regarding each item. For each question there was a choice between 7 answers, each corresponding to a numerical value ranging from − 3 to + 3, “0” meaning “non influence”. These 14 questions were similar for all the groups.

**Table 1:** Multiple choice questionnaire distributed to all the participants

1) According to you, how will the DCR influence the *health care access of PWUD*?						
A lot less health care access (− 3)	Less health care access (− 2)	A bit less health care access (− 1)	No influence (0)	A bit more health care access (+ 1)	More health care access (+ 2)	A lot more health care access (+ 3)
2) According to you, how will the DCR influence the *frequency of drug use of PWUD*?						
A lot less drug use (− 3)	Less drug use (− 2)	A bit less drug use (− 1)	No influence (0)	A bit more drug use (+ 1)	More drug use (+ 2)	A lot more drug use (+ 3)
3) According to you, how will the DCR influence the risk of *injection-related infectious diseases*?						
A lot less infections (− 3)	Less infections (− 2)	A bit less infections (− 1)	No influence (0)	A bit more infections (+ 1)	More infections (+ 2)	A lot more infections (+ 3)
4) According to you, how will the DCR influence the risk of *death by overdose*?						
A lot less deaths (− 3)	Less deaths (− 2)	A bit less deaths (− 1)	No influence (0)	A bit more deaths (+ 1)	More deaths (+ 2)	A lot more deaths (+ 3)
5) According to you, how will the DCR influence the *social vulnerability of PWUD*?						
A lot less vulnerable (− 3)	Less vulnerable (− 2)	A bit less vulnerable (− 1)	No influence (0)	A bit more vulnerable (+ 1)	More vulnerable (+ 2)	A lot more vulnerable (+ 3)
6) According to you, how will the DCR influence the *sharing of used smoking and injection devices*?						
A lot less sharing (− 3)	Less sharing (− 2)	A bit less sharing (− 1)	No influence (0)	A bit more sharing (+ 1)	More sharing (+ 2)	A lot more sharing (+ 3)
7) According to you, how will the DCR influence the amount of *new PWUD*?						
A lot less new PWUD (− 3)	Less new PWUD (− 2)	A bit less new PWUD (− 1)	No influence (0)	A bit more new PWUD (+ 1)	More new PWUD (+ 2)	A lot more new PWUD (+ 3)
8) According to you, how will the DCR influence the number of *arrests for drug use*?						
A lot less arrests (− 3)	Less arrests (− 2)	A bit less arrests (− 1)	No influence (0)	A bit more arrests (+ 1)	More arrests (+ 2)	A lot more arrests (+ 3)
9) According to you, how will the DCR influence the *quietness of the neighborhood*?						
A lot less quiet (− 3)	Less quiet (− 2)	A bit less quiet (− 1)	No influence (0)	A bit quieter (+ 1)	Quieter (+ 2)	A lot quieter (+ 3)
10) According to you, how will the DCR influence the *amount of injection or smoking devices abandoned* in the public space?						
A lot less abandoned devices (− 3)	Less abandoned devices (− 2)	A bit less abandoned devices (− 1)	No influence (0)	A bit more abandoned devices (+ 1)	More abandoned devices (+ 2)	A lot more abandoned devices (+ 3)
11) According to you, how will the DCR influence the importance of *drug dealing* in the neighborhood?						
A lot less drug dealing (− 3)	Less drug dealing (− 2)	A bit less drug dealing (− 1)	No influence (0)	A bit more drug dealing (+ 1)	More drug dealing (+ 2)	A lot more drug dealing (+ 3)
12) According to you, how will the DCR influence the *use of drug in public* in the neighborhood?						
A lot less drug use in public (− 3)	Less drug use in public (− 2)	A bit less drug use in public (− 1)	No influence (0)	A bit more drug use in public (+ 1)	More drug use in public (+ 2)	A lot more drug use in public (+ 3)
13) According to you, how will the DCR influence the risk of *violence* in the neighborhood?						
A lot less violence (− 3)	Less violence (− 2)	A bit less violence (− 1)	No influence (0)	A bit more violence (+ 1)	More violence (+ 2)	A lot more violence (+ 3)
14) According to you, how will the DCR influence the *global health and quality of life of PWUD*?						
Health a lot worst (− 3)	Worst health (− 2)	Health a bit worst (− 1)	No influence (0)	Health a bit better (+ 1)	Better health (+ 2)	Health a lot better (+ 3)

*Abbreviations*: *DCR* drug consumption room, *PWUD* people who use drugs

Italic prints: *p* < 0.05

The study design was approved by the INSERM IRB (CEEI-IRB00003888) on July 2016.

The answers to the questionnaire were organized in four categories, describing the potential impact of the opening of the DCR on the following: the health of PWUD, the social welfare of PWUD, an effect of trivialization and incitement of drug use, and the effect on drug-related public disorders.

We created three sets of questions assessing the social and professional characteristics of the GP, of the health professionals that were not GPs, and of the drug users. GPs were asked about their age, their sex, the district in which they worked, the prescription of opioid substitution treatment (OST) in their practice or not, and their experience in managing patients with substance use disorders. The other health professionals were asked about their age, their sex, the district in which they worked, their past experience in the field of addiction medicine in years, and their occupation (pharmacist, emergency doctor, or worker in an addiction care or risk reduction facility (including doctor, nurse, social worker, counselor, director)). Because most of those professionals were not medical doctors, the question of OST prescription was not evaluated in this group. PWUD’s characteristics were not analyzed in this study.

We contacted by phone every GP and pharmacy listed in the phone book as working in the 9th, 10th, or 18th district at the beginning of the summer 2016. If they were interested in the study, we offered to send them an email with a link directing to the online questionnaire and an information form summarizing the objectives and the method of the study, or to fax them these documents. We contacted the head of the emergency department in Lariboisière hospital, who forwarded an email with a link directing to the online questionnaire and an information form to every doctor working in this ER. The heads of every care or harm reduction facility of the 9th, 10th, 18th, and 19th districts of Paris were contacted by phone and/or email. If they agreed to it, the main investigator came to present the study during a staff meeting and ask the willing members of the staff to fill up a printed questionnaire. In the willing harm reduction facilities, anonymous questionnaires and information forms were also left for the PWUD who attended them. The local staff was trained to the study requirement. They were in charge of the information on the study, its purpose, and its anonymity and offered PWUD to participate. The interested PWUD could read the information form and complete the questionnaire alone or with the help of a staff member. Since there was no obligation to participate to the study, filling up the questionnaire amounted to consent. The main investigator collected the completed questionnaires before the opening of the DCR too. Responses were anonymous in all groups.

The statistical analysis was done with SPSS 21.0. For the 14 questions regarding the DCR, the medians and the means of the numerical answers of each group are described (the median was considered as the main result). For the two groups of GPs and other health professionals, univariate comparisons were performed to test the association of the collected variables and the expectations. Because normality assumptions were not always met, we choose to perform only non-parametric tests (Mann-Whitney *U*, and Kruskal-Wallis tests or Spearman’s correlation tests as appropriate).

## Results

We conducted the study during the summer 2016, before the opening of the Parisian DCR in October 2016. Out of the 251 GP of the target population, 119 accepted to receive a questionnaire and 62 effectively participated in the study (24.7%), they formed the first group. Out of the 162 pharmacies of the territory of the study, 72 accepted to receive questionnaires and 30 pharmacists completed them. Out of the 29 doctors working in the ER of Lariboisière hospital, 5 completed a questionnaire (17.5%). Out of the 14 harm reduction facilities of the north of Paris, 9 accepted to participate to the study, of which 45 workers completed the questionnaire. The latter three constituted the group of “other health workers” (*N* = 82). Finally, 57 PWUD from those 9 harm reduction facilities completed a form.

### Results among GPs

The median answers in the GPs were that the DCR would improve the health of PWUD (decrease of the sharing of smoking and injection devices, of the deaths by overdose, and of the drug-related infections, and improvement of the access to health care and global health of PWUD). They also thought that the DCR would have no influence on the access to social welfare for PWUD, that it would not have an effect of trivialization and incitement of drug use (no influence on the number of new PWUD and on the frequency of drug use). Their opinion was mixed regarding the impact on drug-related public disorders (they thought that the DCR would decrease the tranquility of the neighborhood, but would have no influence on the violence, the number of arrests for drug use, or the drug dealing, and would result in a decrease of drug consumption in the public space and of the amount of abandoned injection and smoking devices) (see Table 2).

**Table 2:** Results of the expectation questionnaire for the three groups (means; standard deviation)

Influence of the DCR	Opinion of the GP (*N* = 62) (median; *mean*)	Opinion of the other health professionals (*n* = 82) (median; *mean*)	Opinion of the PWUD (*N* = 57) (median; *mean*)
Health care access of PWUD	A bit more (1; *0.85*)	A bit more (1; *1.15*)	More (2;1.*25*)
Global health an quality of life of PWUD	Health a bit better (1; *0.85*)	Health a bit better (1; *1.24*)	Health a bit better (1; *1.19*)
Sharing of used consumption device between PWUD	Less (−2; *−1.50*)	Less (− 2; *− 1.86*)	A lot less (−3; *− 2.09*)
Risk of drug-related infection	Less (− 2; *− 1.73*)	Less (− 2; *− 1.98*)	Less (− 2; *− 1.96*)
Risk of death by overdose	A bit less (− 1; *− 1.15*)	Less (− 2; *− 1.52*)	Less (− 2; *− 1.79*)
Social disadvantages of PWUD	No influence (0; *− 0.39*)	A bit less (− 1; − *0.68*)	No influence (0; *− 0.61*)
Frequency of drug use	No influence (0; *0.35*)	No influence (0; *0.10*)	No influence (0; *0.25*)
Number of new PWUD	No influence (0; *0.21*)	No influence (0; *0.14*)	No influence (0; *0.11*)
Quietness of the neighborhood	A bit less (− 1; *−1.03*)	No influence (0; *− 0.30*)	No influence (0; *0.28*)
Amount of drug dealing in the neighborhood	No influence (0; *0.31*)	No influence (0; *0.10*)	No influence (0; − *0.07*)
Number of arrests for drug use	No influence (0; *0.27*)	No influence (0; *− 0.09*)	A bit less (− 1; − 0.55)
Violence in the neighborhood	No influence (0; *0.23*)	No influence (0; − *0.35*)	A bit less (− 1; − 0.87)
Amount of consumption devices abandoned in the public space	A bit less (− 1; *− 1.07*)	Less (− 2; *− 1.59*)	Less (− 2; *− 1.77*)
Drug consumption in the public space	A bit less (− 1; *− 0.84*)	Less (− 2; *− 1.39*)	Less (− 2; *− 1.39*)

The univariate analysis revealed that age, sex, or district of exercise were not significantly associated with a different expectation. Reversely, GPs who declared that they had experience in addiction medicine, with the longer duration of this experience, and that they currently prescribed OST had significantly higher expectancies that the DCR would improve the access to social rights of PWUD (respectively MW *p* = 0.019, Spearman’s rho *p* = 0.009 and MW *p* = 0.030) and their health condition (respectively MW *p* = 0.004, Spearman’s rho *p* = 0.021 and MW *p* = 0.002) (see Table 3).

**Table 3:** Univariate analysis of factors associated with GPs’ answers (*N* = 62)

	Age (Spearman’s rho)	Sex F = 29 M = 32 (MW)	District of exercise (KW)	Experience in addiction medicine Yes *N* = 24 No *N* = 36 (MW)	Years of practice of addiction medicine (Spearman’s rho)	Prescription of OST Yes *N* = 19 No *N* = 43 (MW)
Health care access of PWUD	*r* = 0.119 *p* = 0.373	*U* = 462 *p* = 0.976	KW = 0.902 *p* = 0.825	*U* = 390 *p* = 0.512	*r* = 0.102 *p* = 0.438	*U* = 326 *p* = 0.242
Frequency of drug use	*r* = 0.027 *p* = 0.840	*U* = 412 *p* = 0.419	KW = 3.333 *p* = 0.343	*U* = 358 *p* = 0.055	*r* = − 0.204 *p* = 0.117	*Yes* − *0.26 (±0.73)* *No 0.64 (±1.35)* *U = 233* *p = .006*
Risk of drug-related infection	*r* = 0.185 *p* = 0.165	*U* = 383 *p* = 0.221	KW = 0.096 *p* = 0.992	*U* = 362 *p* = 0.244	*r* = − 0.102 *p* = 0.439	*U* = 337 *p* = 0.313
Death by overdose	*r* = 0.130 *p* = 0.329	*U* = 337 *p* = 0.060	KW = 3.087 *p* = 0.378	*U* = 362 *p* = 0.281	*r* = − 0.124 *p* = 0.345	*U* = 347 *p* = 0.406
Social disadvantages of the PWUD	*r* = 0.010 *p* = 0.938	*U* = 378 *p* = 0.191	KW = 6.604 *p* = 0.086	*Yes − 0.79 (± 1.10)* *No − 0.17 (± 1.10)* *U* = 284 *p = 0.019*	*r* = − 0.333 *p = 0.009*	*Yes − 0.79 (± 1.18)* *No − 0.19 (± 1.08)* *U* = 267 *p = 0.030*
Sharing of used consumption devices	*r* = 0.069 *p* = 0.606	*U* = 357 *p* = 0.107	KW = 4.038 *p* = 0.257	*U* = 408 *p* = 0.707	*r* = − 0.038 *p* = 0.774	*U* = 373 *p* = 0.679
Amount of new PWUD	*r* = − 0.046 *p* = 0.736	*U* = 425 *p* = 0.661	KW = 0.353 *p* = 0.950	*U* = 342 *p* = 0.146	*r* = − 0.176 *p* = 0.182	*U* = 299 *P* = 0.081
Number of arrests for drug use	r = − 0.102 *p* = 0.453	*U* = 426 *p* = 0.903	KW = 4.801 *p* = 0.187	*U* = 313 *p* = 0.149	*r* = 0.214 p = 0.107	*U* = 340 *p* = 0.767
Quietness of the neighborhood	*r* = 0.231 *p* = 0.083	*U* = 388 *p* = 0.357	KW = 3.830 *p* = 0.280	*U* = 367 *p* = 0.408	*r* = 0.135 *p* = 0.308	*U* = 314 *p* = 0.220
Amount of drug consumption devices abandoned in public	*r* = − 0.097 *p* = 0.471	*U* = 361 *p* = 0.185	KW = 1.388 *p* = 0.708	*U* = 355 *p* = 0.348	*r* = 0.137 *p* = 0.300	*U* = 339 *p* = 0.521
Deal in the neighborhood	*r* = 0.229 *p* = 0.084	*U* = 452 *p* = 0.863	KW = 0.178 *p* = 0.981	*U* = 359 p = 0.257	*r* = 0.093 *p* = 0.481	*U* = 351 p = 0.438
Drug use in public	*r* = 0.150 *p* = 0.265	*U* = 378 *p* = 0.199	KW = 1.788 *p* = 0.618	*U* = 406 *p* = 0.692	*r* = − 0.038 *p* = 0.773	*U* = 356 *p* = 0.495
Violence in the neighborhood	*r* = − 0.170 *p* = 0.203	*U* = 379 *p* = 0.275	KW = 1.810 *p* = 0.613	*U* = 371 *p* = 0.429	*r* = − 0.127 *p* = 0.336	*U* = 372 *p* = 0.777
Global health and quality of life of the PWUD	*r* = 0.011 *p* = 0.935	*U* = 384 *p* = 0.193	KW = 6.589 p = 0.086	*Yes 1.29 (± 0.80)* *No 0.58 (± 1.13)* *U* = 259 *p = 0.004*	*r = 0.298* *p = 0.021*	*Yes 1.42 (± 0.69)* *No 0.62 (± 1.10)* *U = 221* *p = 0.002*

*GP* general practitioner, *OST* opioid substitution treatment prescription, *MW* Mann-Whitney’s *U* test, *r* Spearman’s correlation test, *P* statistical significance

Italic prints: *p* < 0.05

### Results among other health professionals

The median expectation in the group of other health professionals was that the DCR would improve the health of the PWUD (increase their access to the health care system, decrease at-risk behaviors such as sharing of smoking and injection devices, and decrease both the risk of overdose deaths and drug-related infections, resulting in an overall improvement of the global health of the PWUD). The median expectation in this group was that the DCR would have no effect in the access of PWUD to social welfare and that the DCR would have no effect of trivialization and incitement of drug use. The median expectation in this group was also that the DCR would have no effect on drug-related public disorders (tranquility of the neighborhood, violence, number of drug-related arrests) but would have positive effects on other drug-related nuisance (decrease of drug consumption in the public space and of the amount of abandoned smoking and injection devices) (see Table 2).

Inside this group, the univariate analysis revealed a higher heterogeneity than in the group of GPs (see Table 4).

**Table 4:** Univariate analysis of factors associated with other health professionals’ answers (*N* = 82)

	Age (Spearman’s rho)	Sex F = 47 M = 32 (MW)	District of exercise 9th N = 8 10th *N* = 36 18th *N* = 25 19th *N* = 11 (KW)	Experience in addiction medicine Yes *N* = 53 No *N* = 22 (MW)	Years of practice of addiction medicine (Spearman’s rho)
Health care access of PWUD	*r* = − 0.127 *p* = 0.266	F, 1.40 (± 1.1) M, 0.75 (± 1.5) *U = 527* *p = 0.018*	KW = 5.498 *p* = 0.139	*U* = 461 *p* = 0.136	*r* = 0.032 *p* = 0.781
Frequency of drug use	*r* = 0.077 *p* = 0.504	*U* = 691 *p* = 0.680	9th 1.0 ± 1.29 10th − 0.11 ± 1.0 18th 0.48 ± 1.3 19th − 0.64 ± 1.0 *KW = 9.232* *p = 0.026*	*U* = 453 *p* = 0.175	*r* = − 0.062 *p* = 0.592
Risk of drug-related infection	*r* = − 0.165 *p* = 0.148	*U* = 692 *p* = 0.526	KW = 0.685 *p* = 0.877	*U* = 582 *p* = 0.990	r = − 0.102 *p* = 0.439
Death by overdose	*r* = − 0.146 *p* = 0.204	*U* = 614 *p* = 0.190	KW = 0.730 *p* = 0.866	*U* = 442 *p* = 0.105	*r* = − 0.073 *p* = 0.527
Social disadvantages of the PWUD	*r* = − 0.064 *p* = 0.580	*U* = 701 *p* = 0.600	KW = 5.439 *p* = 0.142	*Yes − 0.79 (± 1.10)* *No − 0.17 (± 1.10)* *U* = 345 *p = 0.004*	*r = − 0.260* *p = 0.022*
Sharing of used consumption devices	*r* = 0.063 *p* = 0.584	F, − 2.38 (± 0.8) M, − 1.06 (± 1.8) *U = 410* *p = < 0.001*	KW = 2.735 *p* = 0.434	*U* = 558 *p* = 0.758	*r* = − 0.021 *p* = 0.858
Amount of new PWUD	*r* = − 0.103 *p* = 0.370	*U* = 718 *p* = 0.688	KW = 4.344 *p* = 0.227	*U* = 486 *p* = 0.177	*r* = − 0.137 *p* = 0.231
Number of arrests for drug use	*r = − 0.255* p = 0.025*	F, − 0.15 (± 1.0) M, − 0.47 (± 1.0) *U = 504* *p = 0.009*	KW = 3.632 *p* = 0.304	*U* = 450 *p* = 0.113	*r* = − 0.198 p = 0.084
Quietness of the neighborhood	*r* = − 0.037 *p* = 0.746	*U* = 692 *p* = 0.542	KW = 6.259 *p* = 0.100	*Yes 0.00 (± 1.53)* *No − 1.32 (± 1.61)* *U = 321* *p = 0.002*	*r = 0.231* *p = 0.042*
Amount of drug consumption devices abandoned in public	*r* = − 0.061 *p* = 0.593	*U* = 715 *p* = 0.705	KW = 2.428 *p* = 0.488	*U* = 479 *p* = 0.209	*r* = − 0.120 *p* = 0.297
Deal in the neighborhood	*r* = 0.000 *p* = 0.997	*U* = 746 *p* = 0.950	KW = 5.794 *p* = 0.122	*U* = 428 *p* = 0.060	*r* = − 0.069 *p* = 0.547
Drug use in public	r = 0.032 *p* = 0.783	*U* = 695 *p* = 0.668	KW = 4.612 *p* = 0.203	*Yes − 1.52 (± 1.09)* *No − 0.86 (± 1.16)* *U = 390* *p = 0.026*	*r* = − 0.218 *p* = 0.057
Violence in the neighborhood	*r* = − 0.078 *p* = 0.503	*U* = 668 *p* = 0.481	9th 0.75 ± 1.16 10th − 0.37 ± 1.26 18th − 0.40 ± 1.19 19th − 1.00 ± 0.77 *KW = 9.090* *p = 0.028*	*Yes* *− 0.57 (± 1.11)* *No 0.36 (± 1.12)* *U = 347* *p = 0.005*	*r = − 0.257* *p = 0.024*
Global health and quality of life of the PWUD	*r* = − 0.116 *p* = 0.317	*U* = 680 *p* = 0.579	9th 1.0 ± 0.75 10th 1.36 ± 0.59 18th 0.92 ± 1.13 19th 1.73 ± 0.64 *KW = 8.247* *p = 0.041*	*U* = 461 *p* = 0.131	*r* = 0.131 *p* = 0.258

*MW* Mann-Whitney’s *U* test, *KW* Kruskal-Wallis test, *r* Spearman’s correlation test, *p* statistical significance

Italic prints: *p* < 0.05

Younger professionals expected a significantly higher decrease in drug-related arrests. Female professionals expected significantly more improvement of health care access for PWUD (MW *p* = 0.018), a higher reduction in syringe sharing (MW *p* < 0.001) but less decrease in drug-related arrests (MW *p* = 0.009) (see Table 4).

In this group, the district of exercise was associated with different expected outcomes of the DCR. Professionals of the 9th district, a little bit farther from the open drug scene and currently less exposed to drug-related nuisance, were the only ones to expect an increase in the frequency of drug use (KW *p* = 0.026) and drug-related violence (KW *p* = 0.028) in their neighborhood. The expectation toward a global improvement of the health and well-being of PWUD was also heterogeneous, professionals from the 9th (less concerned) and 18th district (very concerned by open drug use, including a crack cocaine open drug scene) having less positive expectations than professionals working in the 10th and 14th districts (KW, *p* = 0.041).(see Table 4).

Lastly, we observed that a previous experience in addiction medicine and the duration of this experience was associated with significantly different expectation in this group of other health professionals. Professionals declaring to be experienced in addiction medicine had higher expectations that the DCR would improve the access to social welfare for PWUD (MW *p* = 0.004), but the higher for those with the fewer years of experience (Spearman’s rho, *p* = 0.022). Furthermore, professionals with an experience in addiction medicine had higher expectations that the DCR would not disturb the quietness of the neighborhood (MW, *p* = 0.002), and this expectation was higher for those with the most years of experience (Spearman’s rho, *p* = 0.042). Finally, the professionals with experience in addiction medicine had significantly higher expectations that the DCR would decrease drug use in the public space (MW, *p* = 0.026) and violence in the neighborhood (MW, *p* = 0.005), and for the latter, this was associated with a longer duration of their experience in the field (Spearman’s rho, *p* = 0.024). (see Table 4).

Among this group of other health professionals, the workplace had a major impact on expectations of the outcome of the opening of a DCR. Professionals working in addiction medicine care or harm reduction centers had significantly higher positive expectances toward the DCR to 8 out of 14 items of the questionnaire than pharmacists, while the medical doctors from the adjacent emergency department had intermediate opinions (see Table 5).

**Table 5:** Mean expectation scores depending on the workplace in the group of other health professionals (*N* = 82)

*(Mean of the answers and CI 95%)*	Harm reduction professionals *N = 45*	ER doctors *N = 5*	Pharmacists *N = 30*	
Health care access of PWUD	1.62 [1.40; 1.85]	1.40 [0.72; 2.08]	0.40 [−0.24; 1.04]	*KW = 11.70* *p = 0.003*
Frequency of drug use	− 0.33 [− 0.68; 0.01]	0.20 [− 0.36; 0.76]	0.76 [0.31; 1.21]	*KW = 11.62* *p = 0.003*
Risk of drug-related infection	− 2.16 [− 2.49; − 1.82]	− 2.00 [− 2.88; − 1.12]	− 1.70 [− 2.22; − 1.18]	KW = 2.33 *p* = 0.312
Death by overdose	− 1.73 [− 2.04; − 1.41]	− 1.00 [− 1.88; − 0.12]	− 1.30 [− 1.64; −.96]	KW = 6.82 *p* = 0.033
Social disadvantages of the PWUD	− 0.98 [− 1.74; − 1.30]	− 0.80 [− 1.84; 0.24]	− 0.20 [− 0.56; 0.16]	*KW = 10.74* *p = 0.005*
Sharing of used consumption devices	− 1.73 [− 2.24; − 1.23]	− 2.00 [− 3.24; − 0.76]	− 2.03 [− 2.51; − 1.56]	KW = 0.37 *p* = 0.830
Amount of new PWUD	0.02 [− 0.28; 0.33]	0.40 [− 0.71; 1.51]	0.27 [0.03; 0.51]	KW = 5.30 *p* = 0.070
Number of arrests for drug use	− 0.18 [− 0.53; 0.17]	0.00 [− 0.39; 0.39]	− 0.18 [− 0.53; 0.17]	KW = 1.04 *p* = 0.594
Quietness of the neighborhood	0.42 [− 0.01; 0.85]	− 1.20 [− 3.24; 0.84]	−1.23 [− 1.77; − 0.70]	*KW = 20.091* *p < 0.001*
Amount of drug consumption devices abandoned in public	− 1.84 [− 2.19; − 1.50]	− 1.20 [− 1.76; − 0.64]	− 1.27 [− 1.79;− 0.74]	*KW = 6.33* *p = 0.042*
Deal in the neighborhood	− 0.16 [−0.52; 0.21]	1.00 [− 0.24; 2.24]	0.33 [− 0.15; 0.82]	KW = 5.62 *p* = 0.060
Drug consumption in the public space	− 1.77 [− 2.09; − 1.45]	− 1.20 [− 1.76; − 0.64]	− 0.87 [− 1.30; − 0.43]	*KW = 12.603* *p = 0.002*
Violence in the neighborhood	− 0.86 [− 1.20; − 0.53]	0.80 [− 0.24; 1.84]	0.20 [− 0.21; 0.61]	*KW = 20.51* *p < 0.001*
Global health and quality of life of the PWUD	1.42 [1.17; 1.67]	1.00 [0.66; 1.34]	1.00 [1.00; 1.00]	*KW = 6.12* *p = 0.047*

*PWUD* persons who use drugs, *ER* emergency room, *GP* general practitioners, *KW* Kruskall-Wallis test

Italic prints: *p* < 0.05

### Results among PWUD attending a care or harm reduction center

The responding PWUD’s median expectation was that the DCR would improve the health of attending PWUD (by decreasing the sharing of smoking and injection devices, the number of overdoses-related deaths, and the number of drug-related infectious risk taking and by improving both access to health care system and global health). The median expectation among the groups of responding PWUD was also that the DCR would have no effect on their social welfare. Furthermore, their median expectation was that the DCR would have no effect of trivialization and incitement of drug use and that it would not increase drug-related disorders or drug dealing in the neighborhood. They even expressed that the DCR would decrease the number of drug-related arrests and violence and the number of abandoned smoking and injection devices and also decrease public drug use (see Table 2).

## Discussion

Overall, our results show that the Parisian health care professionals and PWUD from the surroundings of the to-be-created DCR had a positive opinion of its impact, even though its effect on PWUD social rights access was not known or judged ineffective and though the GPs and the pharmacists thought that it could create some mild drug-related public disorders.

The participation of the GP to this study is comparable to most studies involving French GPs. They mainly think that the DCR will have positive effects, which is consistent with the study that showed that 61.5% of the GPs of the north of Paris were in favor of the DCR [24].Their representations of the effects of the DCR is consistent with the DCRs’ objectives and their efficiency as demonstrated in the international literature [1–12, 17, 18], except for the absence of impact on PWUD social rights access. Even though we did not find any study that evaluated the effects of DCRs on the access to social welfare, it is reasonable to expect one given that there is a social worker in the French DCR and that the public health insurance operates on site a basic service to open or extend the rights of the PWUD.

The most favorable expectation were found in GPs with a training and current experience in treating patients with substance use disorders.

Other health professionals had also globally positive expectations of the future DCR. Although this group was much more heterogeneous geographically and in the type of professional position, we could observe also that a previous experience in addiction medicine, or currently working in an addiction care or harm reduction facility, was significantly associated with higher expectations. If the pharmacists were expressing significantly less positive expectations of the DCR, they still thought that it would globally reduce risk taking and improve the health and quality of life of PWUD. This is consistent with the findings of previous studies showing French pharmacists opposing to harm reduction policies, perhaps due to a lack of addiction medicine or harm reduction principles and to a general feeling of isolation in their care of PWUD, but changing in the years of opioid substitution treatment access enlargement in France [32].

The low number of ER doctors who participated makes an interpretation of their answer difficult, and their representativity is questionable, but they seem to have globally a good opinion of the DCR in terms of health improvement for PWUD, but at the same time, and because our study only involved the emergency room directly adjacent to the soon to be opened DCR, they thought that it would increase the number of PWUD in their immediate neighborhood and bring more disorders in the public place. On that point, the opinions collected in our study may seem less favorable than those expressed by ER doctors inquired in other countries [31]. But then the question was asked about a putative opening of a DCR in cities that had no such facility, but our study was as far as we are aware of the only study conducted with the precise location of a DCR opening the very next door of the ER where the respondents are employed.

The responding PWUD had a good opinion of the impact of DCR. Their representations are consistent with the objectives of this structure, which means that they understand well the potential benefit of a DCR. This is in accordance with several other studies conducted in different countries were DCR operate [25–30]. The only exception is their disbelief in its social objective efficacy.

The involvement of PWUD in harm reduction initiatives is essential to ensure harm reduction services reflect their current need, develop trust, and foster their empowerment. [33] Moreover, PWUD have proved that they are able to invest themselves in harm reduction initiatives, and even become influential partners in expanding and improving the local and national harm reduction initiatives [34]. Knowing that they have a positive opinion of the French DCR therefore matters.

The results of this study should be interpreted keeping in mind that it has several limits. First, it would have been more methodologically rigorous to make six separate groups (GP, ER doctor, harm reduction professionals, pharmacists, PWUD) instead of three groups with one “other health professionals” group but we were not sure that we could get enough participants to analyze the answers correctly. Especially, the low number of ER doctors is definitely an obstacle to a correct interpretation of their representations, and other studies should be made to investigate them. Second, there might be a selection bias regarding responding PWUD. Since they were recruited in addiction care/harm reduction facilities, they might be more informed and sensitive to harm reduction, and they might have a better opinion of the DCR than the general population of PWUD in the north of Paris.

Furthermore, because this study was exploratory, we choose not to apply multiple testing corrections to our results and not to calculate the effect size of observed differences. So there is a chance that some results that we observed are false positive.

But our study also has some strength, one of them being that we conducted it when the DCR opening was more than a project but already decided and its future place already known. Furthermore, the opinion of most health professionals regarding DCRs had not been interrogated in France before. Especially the opinion of pharmacists, who are an essential part of the community care system in France, had to our knowledge never been investigated in the international literature. Even though our effectives remain modest, we solicited a large panel of health professionals whose opinions were not known. This study constituted also an opportunity for the Parisian PWUD to express themselves publicly on the controversial subject of the French DCR, making their voice audible being in itself an act of empowerment.

This study was also an opportunity to “take a picture” of the representations of involved people before the opening of an experimental and new structure in France, and it could be interesting to reproduce the same study in a few years to observe if changes occurred in these representation after their confrontation with the real impacts of the French DCR.

## Conclusion

This study shows that shortly before the opening of the first French DCR in Paris, the nearby GPs, pharmacists, ER doctors, harm reduction professionals, and PWUD thought mainly that the DCR would have a positive impact by increasing PWUD’s harm reduction behavior and a positive impact on their general health, and no negative impact in terms of increasing drug use. Both professional and PWUD respondents thought that the DCR would have also a positive impact in terms of reducing some drug-related nuisance in the public space. The opinion of all health professionals was significantly better if they were experienced in addiction medicine.

## Abbreviations

CI 95%: Confidence interval of 95%
DCR: Drug consumption room
ER: Emergency room
GP: General practitioner
HCV: Hepatitis C virus
HIV: Human immunodeficiency virus
MCQ: Multiple choice questionnaire
OST: Opioid substitution treatment
PWUD: People who Use drugs
